# Protocol: The effect of restorative justice interventions for young people on offending and reoffending: A systematic review

**DOI:** 10.1002/cl2.1403

**Published:** 2024-05-15

**Authors:** Hannah Gaffney, Darrick Jolliffe, Elizabeth Eggins, Joana Gomes Ferreira, Guy Skinner, Barak Ariel, Heather Strang

**Affiliations:** ^1^ Clare Hall College University of Cambridge Cambridge UK; ^2^ Institute of Criminology University of Cambridge Cambridge UK; ^3^ Royal Holloway University London UK; ^4^ Metro North Mental Health, Queensland Health Brisbane Queensland Australia; ^5^ Department of Social and Policy Sciences Univeristy of Bath Bath United Kingdom; ^6^ Department of Public Health and Primary Care University of Cambridge Cambridge UK

## Abstract

This is the protocol for a Campbell systematic review. The objectives are as follows. The primary aim of this mixed methods review is to synthesise the available evidence regarding the effectiveness of restorative justice interventions (RJIs) for reducing offending and reoffending outcomes in children and young people. We are also particularly interested in the impact of RJIs on children and young peoples' violent offending and violent reoffending. A second aim of the review is to examine whether the magnitude of effectiveness of RJIs may be influenced by study characteristics such as the population (e.g., age, ethnicity, or sex), the form of intervention (e.g., face‐to‐face mediation compared to family group conferencing), the place of delivery of the intervention (e.g., in independent office, in court), implementation (e.g., trained facilitators, dose, fidelity) and methodology (e.g., randomised controlled trial). The third aim of the review is to synthesise the qualitative evidence about RJ to develop a better contextual understanding of how these programmes may work and to elucidate factors that might increase the efficacy and implementation of RJ interventions. The specific research questions this systematic review aims to address are: (1) Do RJ interventions reduce children and young people's involvement in offending or reoffending relative to a comparison group? [RQ1]. (2) Is there variation in the impact of different RJ approaches on young people's involvement in offending or reoffending? [RQ2]. (3) Is there variation in the impact of RJIs on children and young people's offending or reoffending depending on the characteristics of the participants taking part in the RJI (e.g., sex, age, ethnicity)? [RQ3]. (4) What characteristics of RJIs, influence the effectiveness of RJIs for children and young people's offending and reoffending? [RQ4]. (5) What are the most notable barriers and facilitators, as reported by participants (e.g., the victims, children/young people, or mediators who have taken part in an evaluation of an RJI, or those children or young people who were meant to take part in an evaluation but ultimately did not), to the implementation of RJIs to reduce later offending or reoffending? [RQ5].

## 1 BACKGROUND

### 1.1 The problem, condition or issue

Preventing and responding to the involvement of children and young people in crime and violence is a global public health concern (World Health Organisation, 2020). One prominent approach for children and young peoples' offending and reoffending is Restorative Justice (RJ), which is a unique ideological approach for responding to crimes and other harms. Definitions of RJ vary. Indeed, many researchers have noted that RJ has become an ‘umbrella concept’, with no universally accepted definition (Shapland et al., 2006). In a criminal justice context, definitions of RJ stress the activity of those affected by an offence (i.e., the victim, offender and affected communities) coming together with the aim of dealing with the offence aftermath and restoring the imbalance created by the offence committed.

For example, Marshall (1999) (p. 5) defined RJ as ‘a problem‐solving approach to crime which involves the parties themselves, and the community generally, in an active relationship with statutory agencies’. A practical example of this definition of RJ would be community conferencing in which victims, offenders and supporters of both victims and offenders are led in a facilitated discussion (by a trained facilitator) to consider how the harm caused by the offence can be repaired (e.g., People & Trimboli, 2007).

Other definitions of RJ propose a more expansive scope which is ‘intention‐based’ suggesting that this is ‘every action that is primarily oriented toward doing justice by repairing the harm that has been caused by a crime’ (Bazemore & Walgrave, 1999, p. 48). A practical example of this definition of RJ would be having the offender undertake a form of community service that is related to the target offence. Through this range of practices, however, there is consensus that the goal of RJ is to reduce the harm caused by the offence (Braithwaite, 2002), and its advocates claim that RJ yields benefits of various magnitude for victims, perpetrators and communities (Braithwaite, 2002).

In addition to encompassing a wide range of practices, RJ can also be delivered at different stages of the criminal justice system. This can include diversion from formal criminal justice processing and in parallel to criminal justice processing. In addition, meetings between perpetrators, victims and community members can take place at any point in the criminal justice process ranging from the point of arrest through to pre‐release from prison (Daly, 2002).

#### 1.1.1 What are some of the forms of RJ?

As mentioned, restorative justice is an umbrella concept, thus incorporating a myriad of different possible activities and concepts. Umbreit and Armour (2011) named ‘restorative justice dialogue’ as the most widely used and evidence‐based restorative justice intervention (RJI). Included in this conceptualisation were activities such as victim‐offender mediation, group conferencing, and peacemaking circles, for example.

For example, McCold (2000) outlined a number of forms of RJ and classified these activities based on an estimation of how ‘restorative’ they are (see Table 1). This is by no means an exhaustive list of all possible restorative justice practices, but instead highlights how not all activities that are labelled restorative justice are equal in the degree of ‘restorativeness’. Moreover, this classification of RJ practices is a Westernised representation of practices with a long tradition in Indigenous communities across the world. Some critique this conceptualisation as misrepresentation and appropriation of forms of conflict resolution used by Indigenous peoples such as Maori communities in New Zealand (e.g., Moyle & Tauri, 2016).

**Table 1:** Examples of activities categorised based on degree of restorative justice involved.

Fully restorative	Mostly restorative	Partly restorative
Peace Circles	Victim Support Circles	Victim Services
Family Group Conferencing	Victim Restitution	Victim Crime Compensation
Community Conferencing	Victim‐Offender Mediation	Offender Community Service
	Therapeutic Communities	Youth Aid Panels
	Positive Discipline	Reparative Boards
	Victimless conferences	Victim Awareness Training for the Offender
		Offender Family Services
		Family‐Centred Social Work

*Source*: Adapted from McCold (2000, p.34).

Those typically considered fully restorative (e.g., peace circles, family group conferencing, community conferencing) are those which address the three pillars of RJ, namely addressing the needs of the victims for reparation, offenders for responsibility and the communities of care for relational reconciliation and reintegration (McCold, 2000; Zehr, 1990). One commonly evaluated form of RJ is victim‐offender conferencing (e.g., Strang et al., 2013). In this form of RJ the victim and the offender may begin the process by meeting with a facilitator individually (Zehr, 2002). If both parties agree to meet for conferencing, a trained facilitator will mediate the conversation with the aim of having the offender take responsibility for the harm caused by the offence, find a mutually agreeable resolution, and possibly having the victim receive an apology (Zehr, 2002). Today, such meetings are also facilitated in non‐face‐to‐face forms, such as over the phone or through a third‐party.

There is no clear delineation between the levels of the forms of RJ listed in Table 1. In evaluating the level of ‘restorativeness’ of an intervention it will be important to determine what activities actually take place. Umbreit and Armour (2010) have emphasised the importance of assessing RJIs on this spectrum of ‘restorativeness’, and this is the approach adopted by recent high‐quality reviews of restorative justice with children and young people (i.e., Kimbrell et al., 2022).

RJ is often suggested as being a suitable approach to youth justice, and the aim of the proposed review is to examine the effectiveness of RJIs for children and young people involved in crime and violence. Suzuki and Wood (2018) outline three reasons for this, namely that children usually commit less serious offences than adults and children are perceived to be less culpable than adults. Furthermore, due to the fact that they are children, it is possible they are cognitively and developmentally more amenable to change, especially in terms of the development of empathy and moral reasoning (Suzuki & Wood, 2018). However, the suitability of restorative justice for children and young people has also been challenged and some have suggested it may lead to undesirable effects. For example, children may not have the necessary communication skills to adequately participate in restorative justice conferencing (Rossner, 2013). Therefore, research that can aid our understanding of the effectiveness of RJIs for children and young people involved, or at risk of involvement, in crime and violence is important.

### 1.2 How the intervention might work

Braithwaite (1989) theory of reintegrative shaming is a predominant part of the theory of change involved in RJIs. A recent review of the effectiveness of RJIs for children and young people outlines that reintegrative shaming is the process by which a community can express their ‘disapproval’ of the offender's actions (i.e., the offence they committed) which is followed by the ‘re‐acceptance’ of the offender into the community (Wong et al., 2016, p. 1312). Braithwaite (1989) posits that shame can occur negatively or positively in restorative justice. In the negative sense, Braithwaite refers to stigmatising shame in RJ as that which occurs when an offender internalises the shame and it becomes part of their self‐identity, however, shame can work positively when it is used as a way to sanction the *behaviour* of the offender, but the value of the offender as a human being is affirmed (Braithwaite, 1989; Suzuki & Wood, 2018). However, as Strang (2020) outlined, the ideas that underpin this idea of reintegrative shaming were not new and Braithwaite himself acknowledged that these concepts had been present in Indigenous communities worldwide, including those across Oceania, Asia, Africa, the Middle East and the Americas. This is an important consideration when attempting to understand the theory of change in RJ in a criminal justice context. Whilst not all restorative justice scholars agree that reintegrative shaming is central in this approach, reparation and *reducing* shame is a central goal of some forms of RJIs (e.g., family group conferences; Frost et al., 2012; McGinn et al., 2020; Pitt et al., 2020).

There are also elements of other criminological theories incorporated into RJ. Procedural justice is an important element, in particular elements of fairness and ensuring all participants have an equal opportunity to contribute to proceedings (Daly, 2002). When RJIs are implemented before formal criminal justice processing, there is also a diversionary element (Wong et al., 2016). The beneficial impact of RJ may be partially attributed to the fact that RJ is often used to supplement diversion from the criminal justice system. Diversion from official processing is widely evidenced as an effective way to reduce the offending of young people (Petrosino et al., 2010).

Previous reviews in this area have also suggested that RJ can minimise the labels associated with involvement in the criminal justice system and thus encourage greater prosocial behaviour and promote desistance (e.g., Wong et al., 2016). Strang et al. (2013) also outlined the importance of theories of procedural justice, defiance, and responsive regulation in restorative justice programmes, but emphasised that there is no single theory that fully and appropriately explains the theory of change in restorative justice programmes. Strang et al. (2013) highlighted Collins (2004) theory of interaction ritual chains, in which the presumed causal mechanism is centred on the motivational impact of the intense emotions experienced by participants in RJ conferencing.

However, as previously discussed, restorative justice is an umbrella term, often used to describe many different types of intervention activities. In fact, many advocates of RJ propose that is it the uniqueness of RJ that enables and facilitates impact and change. As Shapland et al. (2006, p. 507) outlines:

…restorative justice, by definition, is created anew each time a set of participants come together to consider that offence and what should happen as a result. So, restorative justice is not a ready‐made package of roles, actions and outcomes that can be plucked off the shelf, but has to be, often quite painfully, made from its basic ingredients by the particular participants who have been brought together as a result of the offence.

Thus, the theory of change involved in RJ is one framed by broader concepts and ideas. Yet, there are key elements which are fundamental to the presumed causal mechanisms in restorative justice. For example, interaction and effective communication between victims and offenders in restorative justice conferencing (Rossner, 2013). Communication specifically about the events leading to the offence and about what should happen in the future are fundamental aspects for RJ (Shapland et al., 2006). Another important factor in RJ is the voluntary nature of the intervention. Victims too have to agree to participate voluntarily, especially as forced participation could lead to further re‐victimisation (Daly, 2002).

It is also possible that the RJ process may have an impact on risk or protective factors related to offending. For example, the intense experience of emotions may increase an offender's level of empathy (Wallis, 2014). Low empathy, or a diminished ability to understand or experience the emotions of others, is commonly implicated as a risk factor for offending (Jolliffe & Farrington, 2021). There is some evidence that, being in the presence of the victim (i.e., where there is a face‐to‐face meeting), the offender is encouraged to appreciate the harm caused by an offence which may lead to increased empathy (Kuehn et al., 2014).

Reparations can work in various ways, which may be deterrence (i.e., having to pay a fine may deter an offender from committing a similar offence in the future) but may also include developing an offenders' self‐worth and a sense of community. Reparations does not necessarily mean financial or material reparations in RJ (Shapland et al., 2006). There is also the concept of ‘symbolic reparation’, which is a fundamental component of RJ. Shapland et al. (2006) outlines that for symbolic reparation to occur, the person (child or adult) must admit remorse for their actions and behaviours, the victim(s) need to perceive this remorse as sincere and be willing to accept an apology and the offer to make amends. However, RJIs can take place without an apology, with recent findings suggesting that an apology, either in full or a partial apology, is included in only half of victim‐offender mediation agreements in the UK (Dhami, 2016).

Alternatively, some have suggested that poorly facilitated RJIs may have a non‐desirable impact. For example, offenders might become frustrated in RJ events, if they were hectored by victims or their own supporters, such that they might become more defiant and re‐offend more thereafter (Sherman, 1993). Suzuki and Wood (2018) have outlined that there are also important considerations to be made about the suitability of RJ for children and young people. Whilst RJ is often perceived as being appropriate for children and young people who have committed an offence, in comparison to adult offenders, children may: (1) not fully comprehend RJ proceedings and may be more vulnerable to suggestion by adult participants; (2) have poorer communication skills; and (3) not be as emotionally mature (Rossner, 2013; Suzuki & Wood, 2018). Therefore, children may not fully understand the voluntary nature of restorative justice and so their motives for participation may hinder outcomes, with some only participating to avoid the alternatives or to protect their reputations (Suzuki & Wood, 2018). Both verbal and non‐verbal communication are vital in achieving ‘symbolic reparation’ in RJ as described previously. Therefore, there may also be some undesirable outcomes for children and young people involved in restorative justice that need to be considered when evaluating its effectiveness.

RJ may also have beneficial impacts for victims and communities. Victims may receive some form of material restitution from the offender (or work in lieu), but evaluations have tended to focus on the potential emotional benefits for victims (e.g., Nascimento et al., 2023; Strang et al., 2013). In their systematic review Strang et al. (2013) found that victims who experienced RJ reported that they felt that the offender was less likely to re‐offend, were significantly more likely to receive an apology and were more likely to feel that this apology was sincere. There was also evidence that victims who experienced RJ were more satisfied with their experience of the criminal justice system and less likely to report that they were dissatisfied (Strang et al., 2013, p. 41). Importantly, victims also reported less desire for revenge (Sherman et al., 2005), which has important implications for breaking the cycle that underpins a significant proportion of violent crime. Victims also generally reported less anger with their offenders, particularly the victims of violent offences. In their systematic review on the psychological impacts of RJ on victims of crime Nascimento et al. (2023) found that victims who participated in RJ showed significant decreases in post‐traumatic stress symptoms and a host of negative emotions (e.g., fear, anger, guilt, anxiety and distress).

### 1.3 Why it is important to do this review

This section will briefly outline previous reviews on RJIs and their impact. Our proposed review will add to the existing literature by including the most recent research and also incorporating a synthesis of qualitative process evaluations.

A recent review of reviews (Gaffney et al., 2022) identified the most recent and relevant systematic reviews (i.e., Kimbrell et al., 2022; Latimer et al., 2005; Livingstone et al., 2013; Strang et al., 2013; Wilson et al., 2017; Wong et al., 2016). The conclusion of this overview of reviews was that overall RJ interventions have a desirable impact on offending and reoffending rates in children and young people. Wong et al. (2016) reported the results of a meta‐analysis of the impact of ‘restorative diversion programmes’ for young people and found there was a desirable impact on reoffending outcomes, but it is not entirely clear what these intervention programmes entailed. Wilson et al. (2017) conducted an extensive systematic review and meta‐analysis of evaluations of restorative justice with children and young people, across a diverse range of programmes. Kimbrell et al. (2022) have since updated this review, and found a desirable overall impact of restorative justice on future delinquent behaviour (*g* = 0.23, 95% confidence interval [CI]: 0.14, 0.32). Searches for this review were conducted up to November/December 2020 (Kimbrell et al., 2022) and so our proposed searches will further update earlier findings by searching for the most recent evaluations. Other reviews have not reported effects separately for children and young people (Latimer et al., 2005; Strang et al., 2013) or need to be updated (Livingstone et al., 2013).

This meta‐review also summarised implementation evidence from the United Kingdom and found that victims and offenders were mostly supportive of RJ but that some practitioners (particularly police officers) reported having reservations (Gaffney et al., 2022). Evidence from process evaluations suggests that many young people found that participating in restorative justice was a ‘wake‐up call’ (Mackie et al., 2014) but some barriers to success included lack of staff training/comprehension, delays between offence and RJ conference, and abuse of power in mediation (Littlechild & Sender, 2010). In addition, there is one meta‐synthesis of qualitative evidence on restorative justice (i.e., Suzuki & Yuan, 2022) but this is not specific to children and young people's experiences. Therefore, our review will also make a substantial contribution to the literature on the effectiveness of restorative justice by synthesising the qualitative evidence from process evaluations of RJIs. To our knowledge, our proposed review will be the first to adopt a mixed methods methodology.

The findings of this review have important implications for policy and practice. Not only will the review update searches to synthesise the most recent available evidence, but our qualitative evidence synthesis of process evaluations will provide meaningful insights into the factors associated with effective implementation of RJIs, and also the possible barriers to implementation. This will assist in the future implementation, evaluation, and generalisability of RJIs with children and young people.

Stakeholders in the UK were consulted in the scoping of this review, and the research questions guiding the review were presented to a stakeholder group in October 2022. This UK‐based group comprised of academics, third sector organisations, and representatives from local authorities who use RJ. However, the importance and reach of the proposed review extends beyond the UK context and the findings will have implications for policymakers, practitioners, and researchers worldwide. For example, our inclusion criteria for process evaluations is not limited to any particular context, country, or continent.

Ultimately, this mixed methods review is intended to not only contribute to our understanding about the effectiveness of RJIs but also inform a Toolkit for practitioners developed by the Youth Endowment Fund. Restorative justice is already included as an intervention strand in this Toolkit (Gaffney et al., 2022) but we aim to use this mixed methods review to update the information and provide both practitioners and policymakers in the UK and across the world with the most recent and relevant evidence.

## 2 OBJECTIVES

The primary aim of this mixed methods review is to synthesise the available evidence regarding the effectiveness of RJIs for reducing offending and reoffending outcomes in children and young people. We are also particularly interested in the impact of RJIs on children and young peoples' violent offending and violent reoffending.

A second aim of the review is to examine whether the magnitude of effectiveness of RJIs may be influenced by study characteristics such as the population (e.g., age, ethnicity, or sex), the form of intervention (e.g., face‐to‐face mediation compared to family group conferencing), the place of delivery of the intervention (e.g., in independent office, in court), implementation (e.g., trained facilitators, dose, fidelity) and methodology (e.g., randomised controlled trial [RCT]). The third aim of the review is to synthesise the qualitative evidence about RJ to develop a better contextual understanding of how these programmes may work and to elucidate factors that might increase the efficacy and implementation of RJ interventions.

The specific research questions this systematic review aims to address are:

1. Do RJ interventions reduce children and young people's involvement in offending or reoffending relative to a comparison group? [RQ1]
2. Is there variation in the impact of different RJ approaches on young people's involvement in offending or reoffending? [RQ2]
3. Is there variation in the impact of RJIs on children and young people's offending or reoffending depending on the characteristics of the participants taking part in the RJI (e.g., sex, age, ethnicity)? [RQ3]
4. What characteristics of RJIs, influence the effectiveness of RJIs for children and young people's offending and reoffending? [RQ4]
5. What are the most notable barriers and facilitators, as reported by participants (e.g., the victims, children/young people, or mediators who have taken part in an evaluation of an RJI, or those children or young people who were meant to take part in an evaluation but ultimately did not), to the implementation of RJIs to reduce later offending or reoffending? [RQ5]

## 3 METHODS

### 3.1 Criteria for considering studies for this review

To address the impact of RJIs on children and young people's involvement in offending, reoffending, and violence we will incorporate the inclusion criteria outlined below. Criteria according to the study population, intervention, comparison group, outcome, and study design (i.e., under a PICOS framework), will be employed to identify relevant impact evaluations that will be used to answer RQ1 to RQ4. Our review of the qualitative evidence (RQ5) will employ a PerSpecTIF framework to guide inclusion criteria (Booth et al., 2019; Noyes et al., 2023). Qualitative studies will be assessed for inclusion based on the perspective, setting, phenomenon, environment, comparison, time, and findings.

#### 3.1.1 Types of studies

We will include studies regardless of publication status for all research questions. While we will place no restrictions on the language of publications, the inclusion of potentially eligible non‐English studies will be subject to resources available for translation. All potentially eligible non‐English studies where an inclusion decision cannot be made or where translation resources prohibit full‐text coding and/or effect size calculation will be provided in a supplementary reference list.

##### Research questions 1 to 4: Intervention effectiveness

We will include any evaluation design where participants are assigned to at least one experimental group (i.e., participants receiving RJI) and one comparison group. Participants may be assigned randomly (e.g., RCTs) or in non‐random manner (e.g., quasi‐experimental designs [QEDs]). For non‐random evaluations to be considered for inclusion, authors must have used a method of allocation that minimises selection bias, for example, by (1) matching the intervention and control group on a pre‐existing risk of reoffending measure or using a statistical matching approach such as propensity score matching; or (2) by reporting sufficient data at baseline to appraise any pre‐existing differences between experimental and comparison groups. We will not exclude studies based on baseline equivalence but do require that this could be assessed for the study to be included in the review. Studies will be included if they measure outcomes either before and after the intervention or only after the intervention. As such, evaluations conducted using a one‐group pre‐post design will be excluded.

Evaluations that employ complex types of analysis (e.g., survival analysis; Gallupe, 2021) will be considered for inclusion in the systematic review, if the study meets all other inclusion criteria. Moreover, evaluations that meet other criteria but use complex evaluation methodologies (e.g., regression discontinuity designs, Miller, 2021) will be considered, but a decision on inclusion will be made on a case‐by‐case basis and possibly in consultation with methodological experts. Our decision will be informed by the inclusion of an adequate control or comparison group and the methodological quality of the procedure used to assign participants to treatment and control groups.

Eligible comparison conditions for this review include no intervention (i.e., treatment‐as‐usual), a waitlist control group, or an alternative non‐RJ intervention. Evaluations which do not have a control or comparison group (i.e., one‐group pretest‐posttest design) will be excluded. We will include evaluations that may have implemented multiple treatment arms in comparison to a single comparison group (e.g., full RJI vs. no treatment comparison and partial RJI vs. no treatment comparison). In such instances, we will include effect sizes for treatment‐control comparisons, but treatment‐treatment comparisons (e.g., effect sizes comparing full RJI vs. partial RJI) are excluded.

##### Research question 5: Barriers and facilitators

To assess the barriers and facilitators to the implementation of RJIs, we will include evaluations that have used qualitative research methods. Existing guidance on qualitative evidence synthesis outlines that inclusion criteria in this area are often more difficult to define (Noyes et al., 2023). Specifically, the number of included qualitative studies in a synthesis should be manageable and sufficient enough to integrate and complement the results of the effectiveness analyses (Noyes et al., 2023).

Therefore, we aim to include only ‘trial siblings’ (Noyes et al., 2023) in our analysis of the qualitative evidence on RJIs. These are studies that use mixed methods to evaluate the impact of a RJI and collect both quantitative and qualitative data from the same participants in the same evaluation. These may have been published alongside the results of an impact evaluation or as a separate publication. If a process evaluation was conducted in addition to an impact evaluation that may not meet our inclusion criteria for the meta‐analysis, for example, due to a lack of control group, we will still include the process evaluation in our qualitative evidence synthesis. Justification for this approach is outlined in further detail in the ‘treatment of qualitative research’ section of the protocol.

We will not include studies where qualitative data was collected completely independently of quantitative impact evaluation data. To be included in our qualitative evidence synthesis, participants must have also been involved in an impact evaluation, or assigned to take part in an impact evaluation, of a RJI.

#### 3.1.2 Types of participants

##### Research questions 1 to 4: Intervention effectiveness

We will include evaluations of RJIs which are delivered to children and young people (aged 10–25 years old) who have been involved in crime or violence or those who have had contact with the criminal justice system and are being diverted from traditional/formal processing. We will also include evaluations of RJIs implemented with children or young people who are labelled by primary evaluations as being ‘at‐risk’ of delinquency or anti‐social behaviour. We will exclude RJIs without a criminal justice focus or that form only one part of a larger intervention model, for example, those aimed at reducing bullying or school exclusion. If studies include both adults and young people, we will only include the study if effects are reported separately for children and young people.

Our age range is purposefully broad (i.e., 10–25 years old) in order to fully encapsulate the effectiveness of RJIs for children and young people. The justifications for this age range are threefold. Firstly, this broad age range will allow us to identify evaluations that included youth and adult samples (e.g., Yeong & Moore, 2020) but who do not report data separately for children and young people, and samples that are predominantly children and young people, but perhaps included some adults and therefore have an overall age range beyond 18–21 years old. In these instances, we will attempt to contact authors of primary evaluations in order to retrieve raw data for children and young people in this sample. Secondly, this broad age range will enable us to include evaluations where an offence was committed by a child or young person (i.e., under 18 years old) but the RJI, and subsequent follow‐ups to measure recidivism, took place after age 18. Finally, this age range will allow for a broader range of included evaluations, as in some contexts (e.g., Scotland) children and young people who are released from custodial settings are eligible for support from the same care system up to their 25th birthday (https://content.iriss.org.uk/youthjustice/sc-liberation.html). Moreover, there is emerging evidence and support for a justice system that addresses the distinct needs of young people aged 18–25 years old (e.g., Transition to Adulthood in the UK). When considering inclusion of evaluations with participants older than 18 years (i.e., the traditional age when a child becomes an adult), the age of the participant at the time of the offence that led to participation in an RJI is the most important criteria.

##### Research question 5: Barriers and facilitators

The same participant inclusion criteria apply for RQ5 as for RQs 1–4. However, in our synthesis of the qualitative evidence, we will also include data from adult participants aligned with the implementation of RJIs with children and young people. For example, intervention facilitators or mediators in RJIs, victims, victim/offender supporters, criminal justice professionals or parents.

#### 3.1.3 Types of interventions

Restorative justice is defined as a process whereby ‘all the parties with a stake in a particular offence come together to resolve collectively how to deal with the aftermath of the offence and its implications for the future’ (Marshall, 1999, p. 37). Our review will focus only on restorative justice that takes place in response to a criminal offence whereby the offender or perpetrator is a child or young person.

Interventions which utilise restorative justice approaches that are classified as fully restorative or mostly restorative (see earlier) will be included. We will base our assessment of the restorativeness of interventions, based on what are considered to be the fundamental core aspects of restorative justice: (1) acknowledging harm caused by one party against another; (2) voluntary participation; (3) emphasis on restoration rather than punishment; and (4) deliberative in nature. Interventions typically considered to fall under the restorative justice umbrella are listed in Table 2, all of which will be included in the review (however, this is not an exhaustive list).

**Table 2:** Eligible types of restorative justice interventions.

Fully restorative	Mostly restorative
Peace Circles	Victim Support Circles
Family Group Conferencing	Victim Restitution
Community Conferencing	Victim‐Offender Mediation
	Therapeutic Communities
	Positive Discipline
	Victimless conferences

We will exclude RJIs that are traditionally considered partly restorative, such as victim services, victim crime compensation, or victim awareness training. Moreover, common practices such as restitution (e.g., financial payments to victim(s)) or community service (e.g., court‐ordered work placements in community serving organisation) will be excluded. This is because, in our view, these practices do not meet our understanding of restorative justice. We will also exclude interventions that are described as being, for example, based on restorative principles or developed in line with restorative justice but that did not actually implement any activities that meet our previously outlined criteria. The intervention criteria apply to all research questions (quantitative and qualitative).

#### 3.1.4 Types of outcome measures

##### Research questions 1 to 4: Intervention effectiveness

Our primary outcomes of interest are related to the involvement of children and young people in crime and violence. As such, we will include evaluations of RJIs that report the impact on outcomes of youth offending and reoffending.

We define offending as any offence committed that leads to receiving a court conviction, caution, reprimand or warning, or being arrested. Offending could include property offences or violent offences, however this is not an exhaustive list. We define violent offending to be a crime in which a victim is harmed by or threatened with violence (National Institute of Justice, 2002). Violent crimes include rape and sexual assault, robbery, assault, and homicide. We define violence as the intentional use of physical force or power, threatened or actual, against oneself, another person, or against a group or community, that either results in or has a high likelihood of resulting in injury, death, psychological harm, maldevelopment or deprivation in line with international recommendations (WHO, 2002). We will also include reoffending outcomes, whereby, an offending outcome represent offences committed by individuals with previous arrests, convictions or charges. Reoffending outcomes may also refer to multiple types of offending (e.g., violent offences or property crime).

These outcomes may include a range of operationalisations, such as the prevalence, frequency, and/or the seriousness of an offence. Evaluations may also report the impact of RJIs using different measures of offending and reoffending (e.g., self‐reported offending, arrests, convictions, and/or imprisonment) and all will be included in our proposed review. Self‐reported offending and official measures (e.g., police records) will be eligible for inclusion.

We will also include evaluations that report the effectiveness of restorative justice on other offending‐related outcomes, such as anti‐social behaviour (e.g., acts that cause or were likely to cause harassment, alarm or distress to one or more persons not of the same household, such as misuse of public space, disregard for community/personal wellbeing, intimidation/harassment, and environmental damage; see Home Office, 2014), delinquency, and/or factors known to be associated with later violence and crime (e.g., aggression, substance use, gang membership), as explicated by the Integrated Cognitive Antisocial Potential (ICAP) theory (Farrington & McGee, 2019). We may also include outcomes relating to children and young people's self‐reported victimisation as these could be indicative of involvement in crime and violence. For example, injury by a weapon, threatened with a weapon or involvement in physical fights, may indicate that a child or young person is involved in crime and violence. These may be included and a decision will be made on a case‐by‐case basis, using the operationalisation of each outcome to inform our decision.

These are our secondary outcomes of interest. Evaluations will not be excluded based on the outcomes they report, except in incidents where only outcomes related to victims are reported. If an evaluation only reports the effectiveness of a RJI for children and young people involved in crime and violence on measures of anti‐social behaviour, for example, this evaluation would be included in our meta‐analysis of the impact of RJIs on anti‐social behaviour.

##### Research question 5: Barriers and facilitators

Process evaluations do not necessarily report information specific to a quantifiable outcome. As such, we will include process evaluations that report information or themes regarding the barriers and facilitators to the implementation of RJIs.

Barriers are defined as those elements or aspects of implementation that prevent or restrict participants' ability to take part in the intervention or reduce the efficacy of the intervention. For example, Shapland et al. (2006) outlined that many of the restorative justice schemes in their research stopped using RJIs in cases where there were multiple offenders. They report that this was due to the observation that where multiple offenders were involved it was easier for an offender to try and mitigate their responsibility and role in the offence, by shifting blame to others involved, during RJ mediation (Shapland et al., 2006). As a key element of RJ is the offender accepting and acknowledging responsibility, this often resulted in a breakdown of the RJI. Therefore, this may be an important barrier to the implementation of RJI with children and young people.

In contrast, facilitators of implementation are elements that promote or increase the efficacy of the intervention. For example, many studies have found that emotion and remorse shown by the offender in RJIs contribute to the success of the intervention, notably important for victim satisfaction (e.g., Harris et al., 2004). Thus, elements of RJIs that allow offenders to demonstrate emotion and remorse for their actions may be a significant facilitator for implementation.

#### 3.1.5 Duration of follow‐up

We aim to include all possible follow‐up time points in evaluations of RJIs with children and young people. Our proposed meta‐analytical model will allow for the inclusion of multiple dependent effect sizes (i.e., time‐points). We will also estimate mean effect sizes for end‐of‐intervention time‐points (i.e., the first data collection timepoint following the cessation of the intervention), and any additional follow‐ups (e.g., 1 year post‐baseline, 2 years post‐baseline).

#### 3.1.6 Types of settings

Our review will include evaluations of RJIs implemented in any setting provided the evaluation meets all other inclusion criteria. No restriction is placed on where the RJI takes place. As such, we expect to include evaluations conducted in criminal justice, community, family, or other non‐public settings. While we will place no limitation on the geographical location of the included studies, we will place restrictions on the language of the studies due to resources available for translation.

We will not include interventions implemented in schools, workplaces, or healthcare settings where an RJI has been implemented to resolve conflict that occurred in those contexts (i.e., not focused on criminal justice context). For example, we would include an evaluation of restorative justice implemented with children involved in crime and violence, who were diverted from traditional criminal processing that took place in school buildings. However, we would not include an evaluation of an RJI that was implemented in schools, by teachers, during school hours to resolve conflicts between pupils and/or staff.

### 3.2 Search methods for identification of studies

This review is proposed as an update of an earlier Campbell Collaboration review of restorative justice (Strang et al., 2013). This review aims to both expand and narrow the previous review inclusion criteria. For example, we will use mixed methods and include process evaluations of RJIs and also include impact evaluations conducted using quasi‐experimental methods to expand the review. We will also include other forms of restorative justice, but will restrict evaluations to those implemented with children and young people. Therefore, rather than update the previous search, we will expand and re‐implement a new systematic search.

In line with recommendations made by Kugley et al. (2017), we will conduct robust and comprehensive searches of subject‐specific and multidisciplinary electronic databases, and also conduct supplementary searches of additional resources to identify unpublished grey literature.

#### 3.2.1 Electronic searches

Search terms have been developed and tested, drawing from the previous Campbell Collaboration review and subsequent reviews of restorative justice. Search terms are grouped into three categories aligning with the population, intervention model, and evaluation terminology (see Table 3). The terms within each category will be combined with Boolean OR and the categories will be combined with Boolean AND. Wherever possible, searches will be conducted across the title, abstract, author‐supplied keywords, and subject indexes. Supporting Information: Appendix 1 contains search syntax for one of the search sources (PsycINFO, Ovid).

**Table 3:** Search terms.

Population	Intervention model	Evaluation/design
adolescen* child* delinquen* juvenile* preteen* ‘pre teen*’ teen* young youth* (school NEAR/3 age)	‘therapeutic communit*’ ‘peace circle*’ ‘family group conferenc*’ ‘community conferenc*’ ‘community group conferenc*’ ‘sentencing circle*’ (harm NEAR/3 (repair* OR reparation*)) (victim* NEAR/3 (conferenc* OR mediat* OR restitut* OR reparation* OR repair* OR ‘support circle*’)) ((restorative*) AND (just* OR mediat* OR conferenc*))	‘comparison condition*’ ‘comparison group*’ ‘control condition*’ ‘control group*’ effective* efficac* evaluat* experiment* interven* ‘matched group*’ pilot* program* ‘propensity score*’ quasi‐experiment* ‘quasi experiment*’ random* RCT service* treat* trial* ‘what works’

Table 4 lists the academic databases and grey literature sources that will be searched for the review.

**Table 4:** Electronic search sources.

Citation source type	Specific search sources	
Academic databases	Cochrane Library Cochrane Central Register of Controlled Trials (CENTRAL) Cochrane Database of Systematic Reviews EBSCO*host* EconLit Criminal Justice Abstracts Social Work Abstracts Violence and Abuse Abstracts Elsevier Scopus Informit Australian Criminology Database (CINCH) Ovid International Political Science Abstracts Joanna Briggs Institute Evidence Based Practice Database PsycINFO PsycEXTRA ProQuest Applied Social Science Index and Abstracts Dissertations and Theses Global Education Resources Information Centre (ERIC) International Bibliography of the Social Sciences National Criminal Justice Reference Service Abstracts Policy File Index ProQuest Central: Criminal Justice, Psychology, Political Science, Public Health, Research Library, Sociology PTSDPubs (previously PILOT) Public Affairs Information Service Index Social Services Abstracts Sociological Abstracts Worldwide Political Science Abstracts Web of Science Book Citation Index – Social Sciences & Humanities Conference Proceedings Citation Index – Social Sciences & Humanities Emerging Sources Citation Index Social Science Citation Index	
Open access and grey literature sources	*Source*	*URL*
	*Restorative justice focused:*	
	Asia Pacific Forum for Restorative Justice	https://www.facebook.com/apfrj/about_details
	Australian Association for Restorative Justice	https://www.aarj.org.au/restorative-justice/
	Canberra Restorative Community	http://www.canberrarestorativecommunity.space
	Centre for Restorative Justice	https://regnet.anu.edu.au/research/centres/centre-restorative-justice-crj
	European Forum for Restorative Justice	https://www.euforumrj.org/en
	International Institute for Restorative Practices	https://www.iirp.edu
	Restorative Justice Exchange	restorativejustice.org
	Restorative Justice Council, UK	https://restorativejustice.org.uk
	Restorative Practices Aotearoa, NZ	https://www.rpa.org.nz
	*Broader youth and/or criminal justice sources:*	
	Association of Chief Police Officers (ACPO), UK^a^	https://web.archive.org/web/20150329070011/ http://www.acpo.police.uk/
	Association of Police and Crime Commissioners	https://www.apccs.police.uk/about-the-apcc/
	Australian Institute of Criminology	https://www.aic.gov.au
	Blueprints for Health Youth Development	https://www.blueprintsprograms.org
	Canadian Police Research Centre^a^	https://web.archive.org/web/20100802005150/ http://www.css.drdc-rddc.gc.ca/cprc/index-eng.asp
	Crime Reduction Toolkit	https://www.college.police.uk/research/crime-reduction-toolkit
	CrimeSolutions	https://crimesolutions.ojp.gov
	DART‐Europe E‐theses Portal	https://www.dart-europe.org/basic-search.php
	Evidence for Policy and Practice Information & Coordinating Centre (EPPI‐Centre)	https://eppi.ioe.ac.uk/cms/
	His Majesty's Inspectorate of Constabulary and Fire & Rescue Services	https://www.justiceinspectorates.gov.uk/hmicfrs/
	Home Office, UK (including Ministry of Justice)	https://www.gov.uk/government/organisations/home-office
	International Association of Chiefs of Police	https://www.theiacp.org
	Jill Dando Institute of Security and Crime Science	https://www.ucl.ac.uk/jill-dando-institute/ucl-jill-dando-institute-security-and-crime-science
	Justice Research and Statistics Association	https://www.jrsa.org
	National Council for Crime Prevention, Sweden	https://www.government.se/government-agencies/the-swedish-national-council-for-crime-prevention/
	National Institute of Justice, US	https://nij.ojp.gov
	Networked Digital Library of Theses and Dissertations	https://ndltd.org
	Office of Juvenile Justice and Delinquency Prevention	https://ojjdp.ojp.gov
	RAND Corporation	https://www.rand.org
	Scottish Institute for Policing Research	https://www.sipr.ac.uk
	Social Care Institute for Excellence (SCIE, including ELSC and Social Care Online)	https://www.scie.org.uk
	Social Science Research Network	https://www.ssrn.com/index.cfm/en/
	The Urban Institute	https://www.urban.org

^a^ Now archived, but website still accessible and searchable.

#### 3.2.2 Searching other resources

We will supplement electronic searches in a range of ways.

We will hand‐search the following topic‐specific journals: *The International Journal of Restorative Justice, Restorative Justice*, and *International Journal of Offender Therapy and Comparative Criminology, Journal of Experimental Criminology*. This list is not exhaustive. We will also identify additional journals in a snowballing approach by examining the journals in which included studies are published.

We will also harvest the reference lists of recent reviews of RJIs (i.e., Kimbrell et al., 2023; Wilson et al., 2017; Wong et al., 2016). Fourth, we will harvest the reference lists of all included studies. Fifth, we will use Google Scholar to conduct forward citation searches with all eligible studies to identify published and unpublished literature that may not have been captured by the previous search steps.

In addition, we will contact prominent authors and organisations in restorative justice research to request information on primary studies that could be potentially integrated into the review. This will be undertaken as the last supplemental search. We will begin with individuals on the advisory panel for this funded review and additional names will be compiled. We will also use a ‘snowball’ sampling technique and request experts to provide information for other relevant individuals in their networks.

### 3.3 Data collection and analysis

#### 3.3.1 Description of methods used in primary research

While we are aware of some experimental evaluations of RJIs (e.g., Shapland et al., 2006), because of the practical challenges of undertaking these we expect to identify relatively few RCTs. Earlier reviews also found a limited number of experimental evaluations of RJIs implemented with children and young people (e.g., Livingstone et al., 2013). As a result, we expect most studies to be quasi‐experimental studies. We anticipate that studies will use individuals as the unit of analysis, but may also allocate clusters of individuals to receive RJIs (see ‘Unit of Analysis’ section for how this will be managed). We expect to find a range of outcomes, including but not limited to: official records of arrests, convictions, reoffending, and self‐reported measures of delinquency, offending, anti‐social behaviour and violence. We expect that most evaluations will report outcomes dichotomously, as a comparison of participants in experimental groups who offended and those who did not offend. Some self‐report measures may use continuous measures. Informed by existing reviews in this area, we expect to find a range of evaluations that were conducted in either community or criminal justice settings (e.g., Kimbrell et al., 2023).

#### 3.3.2 Selection of studies

The process for screening evaluations for inclusion/exclusion will be organised in two stages. All screening will be performed in the reference management software *DistillerSR*. First, we will screen the de‐duplicated search results for potential eligibility on their titles and/or abstracts. The focus of this stage of screening will be to appraise general relevance to restorative justice. Screening at this stage will be undertaken by four review authors (DJ, GS, JMGF, and HG). We will apply a ‘two to exclude’ rule, whereby studies excluded at this stage will require two separate coders to exclude. All three coders will screen studies and any conflicts will be discussed in collaboration with the third independent screener.

Potentially eligible studies retained from the title and abstract will move to the second stage of screening. This stage of the screening will use the full‐text and will be undertaken by four members of the team (DJ, GS, HG, and JMGF) with each study screened independently by two individuals. Any conflicts will be resolved in discussion with the review team. All screening will be undertaken in *DistillerSR*, and each study excluded at the full‐text screening will be assigned a reason for exclusion. We will report information about the percentage of agreement between screeners at both screening stages and we will report results from tests for inter‐rater reliability.

Studies will be appraised using the following exclusion criteria: (1) not an empirical study of an RJI; (2) ineligible participants (i.e., outside 10–25 years); (3) ineligible research design; (4) ineligible outcome measure(s). The latter criteria will only be applied to quantitative impact evaluations (RQs 1–4).

#### 3.3.3 Data extraction and management

Two coders will independently extract data from each included primary evaluation using the coding form presented in Supporting Information: Appendix 2. All coding will be conducted in *DistillerSR*. Coding reliability will be monitored with discrepancies recorded. It will be the responsibility of the principal investigator to resolve any discrepancies that arise. This process will be documented, and an estimation of inter‐rater reliability will be computed and reported in the final review.

#### 3.3.4 Assessment of risk of bias in included studies

We plan to assess the risk of bias for included RCTs using the Cochrane Collaboration's tool for assessing risk of bias (RoB‐2; Sterne et al., 2019). The instrument involves five specific domains, namely: (i) randomisation process; (ii) deviations from the intended intervention; (iii) missing outcome data; (iv) measurement of the outcome; and (v) selection of the report result. Each of these domains will be judged on a 3‐point scale (i.e., low risk, high risk, unclear risk), using the signalling questions in the tool.

Risk of bias assessment for included QEDs will be assessed using the Cochrane Collaboration's Risk of Bias In Non‐Randomised Studies of Interventions tool (ROBINS‐I, Sterne et al., 2016). The ROBINS‐I involves assessment of seven domains, namely: (i) bias due to confounding; (ii) bias in the selection of participants into the study; (iii) bias in measurement; (iv) bias due to departures from intended intervention; (v) bias due to missing data; (vi) bias in measurement of outcomes; and (vii) bias in the selection of the reported results. Each domain includes questions that facilitate the judgement of each single report. Each of these domains will be judged on a 5‐point scale (i.e., low, moderate, serious, critical and no‐information risk), using the signalling questions in the tool.

Assessment of risk of bias for each evaluation will be conducted by at least two authors, with disagreements resolved by discussion with a third member of the review team. Results will be presented in a summary tables and graphs, produced using the macro‐embedded Excel tool provided by the Cochrane Collaboration.

#### 3.3.5 Measures of treatment effect

The data used to estimate effect sizes will be recorded in a specially designed Excel spreadsheet, which is currently being used to extract effect sizes from studies included in the Campbell Evidence and Gap Map of interventions to prevent children and young people involved in crime and violence. Using this spreadsheet, two coders will record raw data (e.g., means, standard deviations, frequencies, sample sizes), study‐reported effects (e.g., regression models, analysis of variance, *d* or odds ratios [ORs]) and use built‐in formulae to estimate the relevant effect size. These formulae are those outlined by Borenstein et al. (2009) and Lipsey and Wilson (2001).

Measures of offending and reoffending are commonly presented as a measure of prevalence (e.g., the proportion of a sample committing an offence). As such, we propose to use ORs as the main metric for offending outcomes. The calculation of ORs will be based on a natural log scale (i.e., LORs) with the aim of maintaining the symmetry of the analysis. As suggested by methodologists (e.g., Borenstein et al., 2009), LORs and the standard error of the logs will be converted back to ORs to facilitate interpretation and comparability. ORs and the 95% CIs will be presented. For the purposes of this review, an ORs significantly greater than 1.0 will represent a desirable impact of RJIs for all outcomes. It follows that an ORs less than 1 will reflect an undesirable impact of RJIs and an ORs that equals 1 will represent a null intervention effect.

It is also possible that the impact of RJIs may be additionally reported as a continuous measure (e.g., the mean number of offences over a set period of time). If presented as such we would use Hedges *g* as the main metric. These will also be presented along with their 95% CIs. We will transform Hedge's *g* effect sizes to OR using the transformation outlined by Lipsey and Wilson (2001). As we plan to include outcomes where a reduction in the outcome is indicative of a desirable impact (i.e., offending or reoffending) and outcomes where an increase is indicative of a desirable impact (i.e., victim satisfaction or empathy), some transformations of effect sizes may be needed. To ensure all effect sizes are reported in a consistent direction in the review, we will transform LORs and Hedge's *g* effects sizes by multiplying by −1 where necessary.

#### 3.3.6 Unit of analysis issues

Unit of analysis issues can arise when the data being analysed is reported for different units than the units used to allocate groups or individuals to experimental and control conditions. For example, an RCT of an RJI for children and young people might randomly assign groups of participants to experimental groups based on geographical location, but present data for individuals. In such cases, we will conduct a sensitivity analysis (see Section 3.3.13).

Another unit of analysis issue relates to clustering. Clusters of individuals tend to share similarities which may require statistical correction to ensure that standard errors, confidence intervals and *p*‐values are accurate. It is unlikely that we will need to adjust for clusters of individuals, as RJIs are most commonly implemented with individuals. However, should we identify clustering in the included studies we will address this following the approach outlined by Higgins et al. (2019).

#### 3.3.7 Criteria for determination of independent findings

We propose to extract all relevant effect sizes from the primary studies that meet our inclusion criteria and as such multiple effect sizes from single evaluations may be included in our meta‐analyses. This may arise when evaluations report multiple outcomes (e.g., the prevalence of offending by type, e.g., violent and non‐violent offending), multiple time periods (e.g., 6‐month follow‐up and 12‐month follow‐up), or the inclusion of multiple treatment arms using the same control group (e.g., full RJI vs. control and partial RJI vs. control). We will code all possible eligible outcomes, time points from primary evaluations, and treatment‐control comparisons. However, if a study includes multiple treatment arms where one or more of the trial arms are not RJIs, these will be excluded from the meta‐analysis. Included treatment‐control comparisons in multi‐arm trials will be clearly specified in the Table of characteristics of included studies.

As we suspect that we are likely to encounter dependent effect sizes both within study reports (i.e., effect sizes from the same sample) and between study reports (i.e., effect sizes from studies conducted by the same research group or in the same region) we will use a correlated and hierarchical effects model (CHE model) with robust variance estimation (RVE) to compute weighted mean effect sizes (Pustejovsky & Tipton, 2022). This approach accounts for a complex nested structure of effect sizes, where effects are nested between larger clusters of studies and also within studies themselves and uses restricted maximum likelihood (REML) estimation of variance components. This model uses inverse weighting that is defined by the specific model (Pustejovsky & Tipton, 2022).

Pustejovsky and Tipton (2022) also suggest that the CHE model is appropriate when the structure of dependent effect sizes is only partially known. However, we will use the decision‐tree outlined by Pustejovsky and Tipton (2022) to choose an appropriate model that best fits the nature of our data. We will also likely find dependency in study reports, whereby authors may produce multiple reports of the same evaluation (e.g., different outcomes, analyses, follow‐up time points in separate reports or dissertations and later published works). We will include all reports of each study and group them under a parent study so that each study is only represented once in each meta‐analysis.

#### 3.3.8 Dealing with missing data

If data needed to estimate an effect size is missing from the primary evaluations, we will make attempts to obtain the missing data from the authors. The corresponding authors of primary evaluations will be emailed to retrieve missing data in the first instance. We will aim to contact authors twice to retrieve missing information. If it is not possible to retrieve data sufficient to estimate an effect size, the evaluation will be excluded from quantitative syntheses, but will be included in the summary of included studies. We may also consult other meta‐analysts who have conducted reviews of restorative justice programmes to request any missing data (Pigott & Polanin, 2020).

For missing information in relation to other codes (e.g., mean age of participants) we will apply ‘infer, initiate, impute’ guideline for addressing missingness (Pigott & Polanin, 2020). This approach outlines that the meta‐analyst should first address missingness by making an educated assumption about the missing data (i.e., infer). For example, if a primary evaluation provides the school grade/year of participants but not their age, the age range of participants can be deduced based on the typical age ranges for each grade. Meta‐analysts can also contact primary authors to seek additional information (i.e., initiate), or use statistical methods for dealing with missingness such as listwise deletion or maximum likelihood (i.e., impute). This approach is not recommended for data needed to estimate effect sizes (Pigott & Polanin, 2020).

#### 3.3.9 Assessment of heterogeneity

We expect that there will be heterogeneity in our proposed meta‐analysis and we will use REML model parameters. Due to the inclusion of dependent effect sizes, heterogeneity will be nested both within clusters of studies and between clusters of studies. In our proposed meta‐analytical model, we will report values for heterogeneity at two levels; level 2 *τ* ^2^ which represents the heterogeneity within‐clusters of effect sizes and level 3 *τ* ^2^ which represents the heterogeneity between‐clusters of effect sizes. In addition to reporting these values of *τ* ^2^ we will also report a multilevel version of *I* ^2^ to quantify the proportion of the total variance that is attributed to non‐sampling error variance (Cheung, 2014; Harrer et al., 2021). We will use the {dmetar} and {ggplot2} packages to compute and plot these values for *I* ^2^.

#### 3.3.10 Assessment of reporting biases

To test reporting biases (or publication bias), funnel plots of standard error (from RVE models) will be produced. Given that the interpretation of funnel plots can be subjective (e.g., Borenstein et al., 2009), we plan the inclusion of additional statistical tests on the potential publication bias (i.e., Egger's regression test for plot asymmetry).

As we plan to incorporate dependent effect sizes in our meta‐analysis of the effectiveness of RJIs, the standard tests for publication bias are inappropriate. Rodgers and Pustejovsky (2021) recently outlined that most of these tests assume that effect sizes are independent, with one effect size included per evaluation. Overall, when dependent effect sizes are used in these tests (e.g., Trim and Fill or Egger's regression) for publication bias, the risk of Type 1 error is increased (Rodgers & Pustejovsky, 2021). Using multiple simulation studies, Rodgers and Pustejovsky (2021) found that when RVE is used, to handle dependence in a meta‐analysis, the rate of Type 1 errors in Egger's regression test for publication bias is not greatly affected.

#### 3.3.11 Data synthesis

Data permitting, we will evaluate the impact of RJIs on eligible outcomes using meta‐analyses. We will compute multiple meta‐analyses to best investigate the effectiveness of restorative justice for children and young people across conceptually grouped outcomes. For example, we propose computing a mean effect size for the following outcomes: (1) all offending outcomes; (2) offence specific outcomes (i.e., violent offending, property offences) outcomes; and (3) reoffending outcomes. A similar approach will be used for other eligible outcomes (i.e., antisocial behaviour, delinquency risk factors), whereby studies will be grouped by conceptually similar outcomes for meta‐analyses. At least two conceptually similar studies will be required for meta‐analysis, otherwise, single standardised effect sizes will be reported.

As previously discussed, it is likely that we will have both hierarchical (between‐study) and correlated (within‐study) structure of dependency amongst estimated effect sizes. Therefore, RVE will be used to allow for the inclusion of dependent effect sizes in the meta‐analysis and we will use the CHE working model outlined by Pustejovsky and Tipton (2022). Meta‐analyses will be computed in *R* using the {metafor} package rma.mv function (Viechtbauer, 2010) and the {clubSandwich} package (Pustejovsky, 2022). If the number of studies to be meta‐analysed is small, we will utilise the ‘CR2’ method for small sample adjustments (Tipton & Pustejovsky, 2015). R script will be included in the technical appendices of the final report.

#### 3.3.12 Subgroup analysis and investigation of heterogeneity

On the condition that we retrieve and include a sufficient number of studies, we will perform analysis to explore the potential role of some specific moderator variables that might explain potential heterogeneity in effect sizes. Based on theory and our knowledge of previous research, we have anticipated a number of potential effect modifiers that should be extracted from the selected studies and coded on the data collection instrument (Supporting Information: Appendix 2).

Firstly, we will code information about participants' demographic characteristics such as participants' sex and ethnicity. Recent analysis has shown that the majority of children and young people who were cautioned or sentenced were identified as male (87%; Youth Justice Board, 2022). Moreover, despite the fact that 70% of children and young people who were cautioned or sentenced during March 2020–21 in England and Wales, were identified as White, Black children were still overrepresented in the criminal justice system (Youth Justice Board, 2022). Thus, both ethnicity and gender are important moderator variables. We will also try to examine differences in the effectiveness of RJIs based on participant age, particularly given recent thought about the suitability of restorative justice for children and young people (Suzuki & Wood, 2018).

We plan to first try to extract as many effect sizes for subgroups of participants based on these characteristics as possible. We hope to then compute individual effect sizes for different groups of participants. However, our expectation is that information about participant demographics, especially ethnicity, will be poorly reported in primary evaluations. Therefore, we plan to create subgroups of evaluations based on the percentages of different demographic groups included in study samples. For example, the percentage of participants identified as male or female or White and Black. Where more than 75% of participants in a study sample belong to one demographic group these evaluations will be labelled as a majority (e.g., majority male or majority female). Evaluations that include between 26% and 74% of a particular group (e.g., 48% male and 52% female) will be labelled as mixed. It follows, that evaluations that include less than 25% of one demographic group, will be labelled as minority (e.g., minority male or minority female). In relation to participant ethnicity, this categorisation is likely to be a crude measure and we may have to collapse groups of different ethnicities into an overall ‘minority ethnicity’ category. We will consider the location of the evaluation before labelling different ethnicities as majority or minority.

Other important moderators are related to the intervention characteristics of RJIs for children and young people that could be a potential source of variability in the estimated impact. As previously discussed, there is no universal definition of RJ, and the term is an umbrella for many different intervention activities. Therefore, we will aim to extract information about intervention activities used for RJIs with children and young people. This includes: facilitator information (e.g., role, training, profession), setting of RJI (e.g., community vs. criminal justice setting), the intervention model (e.g., family group conferencing vs. face‐to‐face mediation), and the degree of restorativeness (e.g., fully restorative vs. partly restorative).

Whilst we will compute an overall mean effect size for offending outcomes, we will also produce separate mean effect sizes for different outcome domains. For example, the type of offence (e.g., violent offences or property offences) and the type of data (e.g., self‐reported offending, official records of arrests, or official records of convictions). Finally, we will also examine whether the effects of RJI vary by research design (i.e., RCTs vs. QEDs). Depending on the numbers of evaluations included and the diversity of the evaluation methodologies, we may be able to assess effect sizes for different types of included quasi‐experiments (e.g., those with propensity score matching and those without). Furthermore, we will aim to compare effect sizes based on the type of comparison condition (e.g., no treatment, treatment as usual, or waitlist control groups).

Moderator analyses will be performed in metafor package for *R* using meta‐regression analyses with RVE.

#### 3.3.13 Sensitivity analysis

Since meta‐analysis involves a wide range of decisions, we will conduct sensitivity analyses to test the robustness of these decisions (Higgins et al., 2019). The use of this technique can contribute to increasing confidence in the pooled effects produced by the analysis. Data permitting, we will examine the change to meta‐analyses by removing studies with high risk of bias. This decision will be based on a number of factors, including the overall number of studies and the number of studies categorised as high risk of bias on each risk of bias criterion.

In the event of outliers accounting for heterogeneity, we will also re‐run analysis with these studies removed to examine the effect on the pooled effect size. It may also be necessary to run sensitivity analyses on the statistical procedures that are utilised to compute effect sizes (e.g., transforming effect sizes), or studies that use different units of analysis and units of allocation. If adjustments are required for the presence of clusters in evaluations, we will conduct a sensitivity analysis for different assumed values of intracluster correlations (Armstrong et al., 2018).

In our proposed meta‐analytical model we will need to compute an assumed variance‐covariance matrix to account for correlated effect sizes in our data (Harrer et al., 2021). To do so, we need to make an assumption about the magnitude of correlation of effect sizes and specify a value for rho using the {clubSandwich} package. As recommended, we will perform sensitivity analyses based on different assumed values of rho.

#### 3.3.14 Treatment of qualitative research

We are undertaking a mixed methods review of the impact of RJIs on violence, offending and reoffending so our review will include qualitative research. Based on recommendations from the Cochrane Handbook (Booth et al., 2019; Noyes et al., 2023) we will use the PerSpecTIF framework to inform our inclusion criteria for process evaluations. Our inclusion criteria for process evaluations are outlined in Table 5.

**Table 5:** Inclusion criteria for qualitative evidence synthesis: PerSpecTIF framework.

Criterion	Application
Perspective	Process evaluations that present qualitative findings from the perspective of children and young people that participated in RJIs will be included or children and young people who were assigned to participate in a RJI but did not. Perspectives of facilitators, mediators, victims, or victim supporters will also be included.
Setting	Process evaluations that present qualitative findings from RJIs that wereimplemented in any setting but involved children and young people at‐risk of or documented as being involved in crime and violence. RJIs may have taken place in police stations, schools, or community settings.
Phenomenon	Process evaluations with qualitative data on the perceptions of children, young people, or adults involved in RJIs to reduce the involvement of children and young people in crime and violence will be included. Process evaluations of RJIs implemented to reduce other behaviours, for example, school bullying or disruptive behaviour at school, will not be included.
Environment	N/A
Comparison	Process evaluations that include views of participants who did not partake in a RJI will be included, but only if the children and young people in the comparison group were processed in the traditional criminal justice system.
Time	Process evaluations that present the views of children and young people after they have completed a RJI will be included. Process evaluations that present the views of children and young people who dropped out or failed to participate in an RJI will also be included, but only if the process evaluation reports findings for successful RJIs.
Findings	Process evaluations that include relevant detail about the barriers and facilitators to implementation in RJIs will be included. Process evaluations that present qualitative findings on theoretical aspects of restorative justice may also be included, but only if the findings relate to the barriers and facilitators involved.

Abbreviation: RJI, restorative justice intervention.

We plan to include only ‘trial siblings’, or in other words, process evaluations that are conducted alongside impact evaluations. These types of process evaluations may have been conducted as part of a mixed‐methods impact evaluation, or as a follow‐up study to an impact evaluation to understand participants' perceptions of the RJI. Trial siblings may have been published in the same report, publication, or article as an impact evaluation, however, this is not a requirement for inclusion. If a study undertook a process evaluation but the findings are reported elsewhere, we will include the additional report in our qualitative evidence synthesis. By including only process evaluations that meet this criteria, we will ensure that the evidence will be able to identify aspects of the optimal implementation of evaluated RJIs. This will allow us to consider the qualitative evidence alongside the quantitative impact of evaluated RJIs. This is true of course for ‘trial siblings’ also, but with the additional quantitative evidence we will be able to consider the impact alongside the qualitative evidence.

We expect that we will find a myriad of rich/poor and thick/thin (Noyes et al., 2023) qualitative studies. ‘Rich’ qualitative data is that which contains a sufficient amount of conceptual detail and ‘thick’ qualitative data has a high level of contextual detail. These are considered preferable over ‘poor’ and ‘thin’ qualitative studies. Therefore, we aim to include rich and thick process evaluations to conduct our qualitative evidence synthesis.

We plan to use a thematic synthesis framework where process evaluations of RJIs for children and young people are grouped based on themes and subthemes. We will record relevant extracts from process evaluations that support the themes and subthemes identified. Our focus is on the barriers and facilitators to implementing restorative justice with children and young people. We will also include perspectives of adults involved in the RJI, for example, parents, mediators, facilitators, victims, victim supporters or police professionals.

##### Qualitative evidence synthesis

Garside (2008) outlines that choosing a method for qualitative evidence synthesis should be informed by several factors. The experience of researchers, the time available to undertake the synthesis, the purpose of the review, and the audience for the review are some examples of factors that should be considered (Garside, 2008).

The purpose of our mixed‐methods review of restorative justice with children and young people is to better our understanding of implementing these interventions. We aim to examine the perceived facilitators and barriers to implementation, specifically looking at the factors that participants report either hindered or aided their participation in restorative justice.

As such, we aim to use a thematic synthesis approach (Thomas & Harden, 2008) to synthesise participants' perceptions of restorative justice. This methodological framework involves a three‐stage process for synthesising qualitative evidence, including coding and the generation of descriptive themes, followed by the development of more analytical themes based on the interpretation of the reviewers (guided by the review questions; specifically, RQ5).

##### Searching and study selection

We plan to include ‘trial siblings’, or in other words, process evaluations of RJIs that have been implemented alongside a quantitative evaluation. We expect that we will find a myriad of rich/poor and thick/thin (Noyes et al., 2023) qualitative studies. ‘Rich’ qualitative data is that which contains a sufficient amount of conceptual detail and ‘thick’ qualitative data has a high level of contextual detail. These are considered preferable over ‘poor’ and ‘thin’ qualitative studies. Therefore, we aim to include rich and thick process evaluations to conduct our qualitative evidence synthesis and will continue to search for and screen studies until we reach a saturated final product (i.e., no new themes or subthemes are being identified).

##### Data extraction

As Thomas and Harden (2008) argue, one of the challenges in the synthesis of qualitative studies is determining what counts as ‘data’ or ‘findings’ (e.g., due to various ways of reporting findings in different studies; see Sandelowski & Barroso, 2002). While different strategies can be employed, we will follow the authors' approach of considering findings ‘to be all of the text labelled as “results” or “findings” in study reports’ or papers (Thomas & Harden, 2008). Once this data has been identified, studies will be uploaded verbatim into the software for qualitative data analysis, NVivo.

##### Thematic synthesis

The thematic synthesis process will begin with coding. Using analytical software, reviewers will begin by coding study findings ‘line‐by‐line’. Codes will be developed inductively from the data, although some might be created in advance (deductively) following our review questions (e.g., barriers, facilitators). This coding process is expected to involve some level of *translation*, that is, ‘taking concepts from one study and recognising the same concepts in another study, though they may not be expressed using identical words’ (Thomas & Harden, 2008). Emerging codes will then be organised and grouped into descriptive themes, a process which will allow the development of a coding tree (including themes, subthemes and codes).

Following these two stages, reviewers will turn their attention to the development of analytical themes. This stage involves going *beyond* the data (i.e., the primary studies) and engaging with some level of interpretation. While controversial (as it is dependent on insight and judgement from reviews), this stage will allow reviewers to potentially develop interpretations and explanations which address the review questions.

##### Quality assessment

We will also perform a quality of assessment of included qualitative studies using the CASP qualitative checklist (Critical Appraisal Skills Programme, 2018). This instrument assesses qualitative evidence using several questions across several domains, such as: (1) clarity of aims and research questions; (2) compatibility of research design for research questions; (3) sampling, recruitment of participants, and form of data collection; (4) application of methods; (5) conceptual depth of the findings; (6) management of deviant cases and provision of alternative interpretations; and (7) reflexivity of the researcher (Noyes et al., 2023). Two coders will independently assess included process evaluations on each of these questions and indicate whether or not evaluations meet the specific criteria.

## CONTRIBUTIONS OF AUTHORS

Please give brief description of content and methodological expertise within the review team. The recommended optimal review team composition includes at least one person on the review team who has content expertise, at least one person who has methodological expertise and at least one person who has statistical expertise. It is also recommended to have one person with information retrieval expertise.

Who is responsible for the below areas? Please list their names:

- Content: Hannah Gaffney, Darrick Jolliffe, Heather Strang, Barak Ariel, Guy Skinner, and Joana Gomes Ferreira
- Systematic review methods: Hannah Gaffney, Darrick Jolliffe, Elizabeth Eggins, Guy Skinner, and Joana Gomes Ferreira
- Statistical analysis: Hannah Gaffney and Guy Skinner
- Qualitative analysis: Joana Gomes Ferreira and Hannah Gaffney
- Information retrieval: Elizabeth Eggins

## DECLARATIONS OF INTEREST

Two of the co‐authors (HS, BA) of this protocol have been involved in a previous systematic review related to restorative justice and have been involved in evaluating restorative justice interventions.

### Preliminary timeframe

Approximate date for submission of the systematic review is 1st May 2024.

## SOURCES OF SUPPORT

### Internal sources

- None, Other

### External sources

- Youth Endowment Fund, UK

## Supporting information

Supporting information.

Supporting information.
