# Supplementary material for: Protocol: The effect of restorative justice interventions for young people on offending and reoffending: A systematic review
Source: Campbell Syst Rev. 2024 May 15;20(2):e1403. doi: 10.1002/cl2.1403 (PMC11096643; doi:10.1002/cl2.1403)
Supplement: Supplementary file 1 — Supporting information. [file CL2-20-e1403-s002.docx]

# Appendices

## Appendix 1. Example systematic search syntax

PsycINFO (Ovid), 1806 to May Week 5 2023

| **#** | **Searches** |
| --- | --- |
| 1 | ("comparison condition*" or "comparison group*" or "control condition*" or "control group*" or effective* or efficac* or evaluat* or experiment* or interven* or "matched group*" or pilot* or program* or "propensity score*" or quasi-experiment* or "quasi experiment*" or random* or RCT or service* or treat* or trial* or "what works").ab,hw,id,mh,ot,ti. |
| 2 | exp Evaluation/ or exp Program Evaluation/ or exp Treatment Effectiveness Evaluation/ or exp Intervention/ or exp Treatment/ |
| 3 | 1 or 2 |
| 4 | (adolescen* or child* or delinquen* or juvenile* or preteen* or "pre teen*" or teen* or young or youth* or (school adj3 age)).ab,hw,id,mh,ot,sh,ti. |
| 5 | exp At Risk Populations/ or exp Adolescent Psychiatry/ or exp Adolescent Behavior/ or exp Adolescent Attitudes/ or exp Adolescent Development/ or exp Adolescent Psychopathology/ or exp Juvenile Delinquency/ or exp Juvenile Justice/ or exp Predelinquent Youth/ |
| 6 | 4 or 5 |
| 7 | ("therapeutic communit*" or "peace circle*" or "family group conferenc*" or "community conferenc*" or "community group conferenc*" or "sentencing circle*" or "justice conferenc*").ab,hw,id,mh,ot,sh,ti. |
| 8 | (harm adj3 (repair* or reparation*)).ab,hw,id,mh,ot,sh,ti. |
| 9 | (victim* adj3 (conferenc* or mediat* or restitut* or reparation* or repair* or "support circle*")).ab,hw,id,mh,ot,sh,ti. |
| 10 | (restorative* and (conferenc* or just* or mediat* or restitut*)).ab,hw,id,mh,ot,sh,ti. |
| 11 | exp Restorative Justice/ |
| 12 | 7 or 8 or 9 or 10 or 11 |
| 13 | 3 and 6 and 12 |

## Appendix 2. Full-text coding form

| ***Code category*** | ***Variable name*** | ***Description*** | ***Response options***  *examples* |
| --- | --- | --- | --- |
| Coding process | DATE | Date of coding | dd/mm/yy |
|  | CODER | Coder Initials | *e.g., HG/DJ* |
| Study identification | STUDY_ID | ID number given to each study | ID_1,2,3 |
|  | AUTHOR | Name of first author | *e.g., Gaffney et al.*  *Jolliffe* |
|  | PUB_DATE | Year of publication | *e.g.* (2022) |
|  | OTHER_REPORTS | List of other related reports, for example, journal article publication of a thesis | Freetext |
|  | IMPACT_AUTHOR | For process evaluations published separately to a related impact evaluation, identify the related impact evaluation | Freetext |
|  | PROCESS_AUTHOR | For impact evaluations published separately to a related process evaluation, identify the related process evaluation | Freetext |
|  | COUNTRY | Country in which evaluation was implemented | UK  USA  Canada  Australia  New Zealand  Other (specify)  Unknown |
|  | PUB_TYPE | Type of publication | Journal  Book/book chapter  Master’s/PhD thesis  Governmental report  Conference proceedings  Charity report  Other (specify) |
|  | SEARCH | How was the study identified? | Electronic database  Hand searches  Reference list  Previous review  Peer/expert suggestion  Author shared |
| **Design: Impact evaluations** | DESIGN_IMPACT | What is the research design used to evaluate the programme? | RCT – pre/post  RCT – post only  QED – pre/post matched  QED - post only, matched  QED – pre/post unmatched  Other (specify) |
|  | DESIGN_ALLOCATE | How were the sample allocated to groups? | Random  Based on assessed criteria  Based on observed criteria  Self-selection  Recommendation  Other (specify) |
|  | QED_MATCH | If a quasi-experimental design was used, what type of matching was used? | Statistical  Based on two or more characteristics  Based on one characteristic  No matching  Other (specify) |
|  | UNIT_ALLOCATE | What units were used to randomly (or not) allocate to conditions? | Individuals  Clusters/groups of participants  Other (specify)  Unknown |
|  | UNIT_ANALYSIS | What was the unit of analysis? | Individuals  Clusters/groups of participants  Other (specify)  Unknown |
|  | CONTROL_TYPE | What type of control group was used? | No treatment  Waitlist  Treatment as usual – official processing  Alternative treatment (not RJ)  Alternative treatment (other RJ)  Other (specify) |
|  | TIME_POST | Time between baseline and post-intervention data collection | Number of months |
|  | TIME_FOLLOWX | Time between baseline and additional follow-ups (after end of intervention) data collection, where X refers to the number of follow-up (e.g., 1, 2, 3) | Number of months |
|  | CONSENT_P | Did the study declare the use of a consent agreement forms for perpetrators? | Yes  No  Unclear, not enough information |
|  | CONSENT_V | Did the study declare the use of a consent agreement forms for victims? | Yes  No  Unclear, not enough information |
|  | CONSENT_SIGNP | Who signed the consent forms for perpetrators? | Perpetrators  Parents/caregivers of perpetrators  Other (specify)  Unclear, not enough information |
|  | CONSENT_SIGNV | Who signed the consent forms for victims? | Victims  Parents/caregivers of victims  Supporter of victim/third party representative  Other (specify)  Unclear, not enough information |
|  | STAT_MODEL | What was the main statistical model the authors used to evaluate the programme? | Freetext |
| **Design: Process evaluations** | DESIGN_PROCESS | What was the research design used to investigate participants' perceptions of the implemenation of the programme? | Trial sibling - at the same time as impact evaluation  Trial sibling - after/follow up to an impact evaluation  Independent process evaluation  Other (specify)  Unclear, not enough information |
|  | PROCESS_DATA | What was the main method used to collect qualitative data? | Semi-structured interviews  Unstructured interviews  Structured interviews  Focus groups  Ethnographic study  Telephone interviews  Mixed  Other (specify) |
|  | PROCESS_ANALYSIS | What was the main approach used to interpret qualitative data? | Freetext |
|  | PROCESS_ALLOCATE | How were the sample for the process evaluation selected? | Freetext |
|  | PROCESS_CONTROL | Was qualitative data collected from a control or comparison group? | Yes  No  Unclear, not enough information |
| **Sample size & attrition** | REFERRAL_TYPE | How were participants referred to the programme? | Probation services  Police  Court  Youth Offending team  Self-referred  Non-CJS referral  Other (specify)  Unknown |
|  | N_BASELINE | Baseline sample size | Number |
|  | N_POST | Sample size at post-intervention time point | Number |
|  | N_FOLLOWX | Sample size at additional follow-up data collection, where X refers to the number of follow-up (e.g., 1, 2, 3) | Number |
|  | ATTRITION_PRESENCE | Was attrition present in the study? | Yes  No  Unclear, not enough information |
|  | ATTRITION_RATE | Record the rate of attrition in the whole sample | % or N of participants lost |
|  | ATTRITION_METHOD | What was the method used for dealing with attrition? | Intention-to-treat (ITT) analysis  Treatment of treated (TOT) analysis  Other (specify)  Unclear, not enough information |
|  | ATTRITION_METHOD2 | If intention-to-treat analysis were performed, what approach was used to deal with missingness? | Listwise deletion  Maximum likelihood  Multiple imputation  Other (specify)  Unclear, not enough information |
| **Sample characteristics** | SEX_P | What was the sex of perpetrators? (provide detailed disaggregated data if available). Use terminology and categorisations provided by primary evaluation. | Freetext |
|  | MEANAGE_P | Mean age and standard deviation of overall perpetrator sample at beginning of intervention | Number in years |
|  | AGERANGE_P | Age range of overall perpetrator sample at beginning of intervention: | Range in years |
|  | ETHNIC_P | Ethnicity of perpetrator (provide detailed disaggregated data if available). Use terminology and categorisations provided by primary evaluation. | Freetext |
|  | SEX_V | What was the sex of victims? (provide detailed disaggregated data if available). Use terminology and categorisations provided by primary evaluation. | Freetext |
|  | MEANAGE_V | Mean age and standard deviation of overall victim sample at beginning of intervention | Number in years |
|  | AGERANGE_V | Age range of overall victim sample at beginning of intervention: | Range |
|  | ETHNIC_V | Predominant ethnicity of victim (provide detailed disaggregated data if available). Use terminology and categorisations provided by primary evaluation. | Freetext |
| **Offence characteristics** | CRIMHIST_P | Do the perpetrators have a criminal history? | Yes  Some, not all  No  Unclear, not enough information |
|  | CRIMHIST_PB | Perpetrators' criminal history was indicated by? | Police contacts  Arrests  Convictions  Incarcerations/imprisonment  Self-reported offending  Unclear, not enough information |
|  | CRIMHIST_PO | Mean and standard deviation of previous offences for perpetrators | Number |
|  | CRIMHIST_V | Do the victims have a criminal history? | Yes  Some, not all  No  Unclear, not enough information |
|  | VOL_P | Did the perpetrator volunteer for the intervention? | Yes  Some, not all  No  Unclear, not enough information |
|  | RESP_P | Did the perpetrators admit  responsibility before the programme? | Yes – voluntary admission  Yes – requirement for participation  Some, not all  No  Unclear, not enough information |
|  | OFFRJ_P | What was the nature of the offence that led to participation in the intervention? | Police contact  Arrest  Conviction  Incarceration  No offence |
|  | OFFRJ_T | What was the type of offence that led to participation in the intervention? | Violent offence  Property offence  Offence against person(s)  Public disorder  Antisocial behaviour  Mutliple offences  Varied between offenders  Other (specify)  Unclear, not enough information |
|  | OFFRJ_Excl | Were any offences excluded from the intervention eligibility? | Violence  Sex offences  Domestic violence/Intimate partner violence  Other (specify)  Unclear, not enough information |
| **Intervention characteristics** | TYPE_RJ | What type of restorative justice was evaluated? Use additional space for providing extra information where necessary | Freetext |
|  | VIC_PREP | Were the victims provided with preparation before the intervention? | Yes  No  Unclear, not enough information |
|  | PERP_PREP | Were the perpetrators provided with preparation before the intervention? | Yes  No  Unclear, not enough information |
|  | MEANTIME_RJ | Mean length of time (and standard deviation) between the offence and the intervention (in days) | Number (in days) |
|  | COMM_PRES | Were members of the community present during or involved in the intervention? | Yes  No  Unclear, not enough information |
|  | COMM_PRES2 | Who were the members of the community present? | Freetext |
|  | VIC_PRES | Were the victims present during the intervention? | Yes  No  Unclear, not enough information |
|  | SUPV_PRES | Were supporters of the victim present during the intervention? | Yes  No  Unclear, not enough information |
|  | SUPP_PRES | Were supporters of the perpetrator present? | Yes  No  Unclear, not enough information |
|  | FACIL_1 | How many facilitators were involved in the intervention activities? | One  More than one  Unclear, not enough information |
|  | FACIL_2 | What was the background of the facilitator(s)? | Independent organisation  Community leader  Police  Psychologist  Social worker  Other (specify)  Unclear, not enough information |
|  | FACIL_3 | Did the facilitator(s) have training provided or previously? | Yes  No  Unclear, not enough information |
|  | RJI_WHEN | When did the RJI take place? | Before official processing (i.e., diversion)  Post-arrest  Post-conviction  Post-incarceration?  Unclear, not enough information |
|  | RJI_WHERE | Where did the RJI take place? | Community (e.g., community centre)  Police station  Probation office  Prison  Other (specify)  Unclear, not enough information |
|  | RJI_EMOT_V | Were emotions expressed by the victim? | Yes  No  Unclear, not enough information |
|  | RJI_EMOT_P | Were emotions expressed by the perpetrator? | Yes  No  Unclear, not enough information |
| **Restorative justice outcomes** | POSTRJI_O1 | Was there an immediate outcome of the intervention (e.g., apology, agreed statement of facts, reparation?) | Yes  No  Unclear, not enough information |
|  | POSTRJI_O2 | What was the immediate outcome of the intervention? | Apology – verbal  Apology - written  Agreed statement of facts  Reparation  Multiple  Other (specify)  Unclear, not enough information |
